# Mapping new pharmacological interventions for cognitive function in Alzheimer’s disease: a systematic review of randomized clinical trials

**DOI:** 10.3389/fphar.2023.1190604

**Published:** 2023-06-01

**Authors:** Inmaculada Xu Lou, Jiayue Chen, Kamran Ali, Abdul Lateef Shaikh, Qilan Chen

**Affiliations:** ^1^ International Education College of Zhejiang Chinese Medical University, Hangzhou, China; ^2^ Department of Cardiology, Hangzhou Hospital of Traditional Chinese Medicine, Hangzhou, China; ^3^ Hangzhou Clinical Medical College Internal Medicine of Traditional Chinese Medicine of Zhejiang Chinese Medical University, Hangzhou, China; ^4^ Department of Oncology, The Fourth Affiliated Hospital, International Institutes of Medicine, Zhejiang University School of Medicine, Yiwu, China; ^5^ Key Laboratory of Combined Multi-Organ Transplantation, Ministry of Public Health, First Affiliated Hospital, School of Medicine, Zhejiang University, Hangzhou, China

**Keywords:** Alzheimer disease, cognition, pharmacology, treatment, latest research, beta-amyloid, immunotherapy

## Abstract

**Background and Objective:** Alzheimer’s disease (AD) is a progressive neurodegenerative disorder, that is, characterized by cognitive decline. To date, there are no effective treatments for AD. Therefore, the objective of this study was to map new perspectives on the effects of pharmacological treatment on cognitive function and the overall psychological state in patients with AD.

**Methods:** Two independent researchers searched for randomized clinical trials (RCTs) exploring new pharmacological approaches related to cognition in Alzheimer’s disease in adults from 2018 to 2023 in PubMed, Web of Science, Scopus, and Cochrane Library databases. A total of 17 RCTs were included in this review.

**Results:** The results show that in recent years, new drugs have been tested in patients with Alzheimer’s disease, including masitinib, methylphenidate, levetiracetam, Jiannao Yizhi, and Huannao Yicong formulas. Most studies have been conducted in populations with mild to moderate Alzheimer’s disease.

**Conclusion:** Although some of the drugs found suggested improvement in cognitive function, the scarcity of available studies highlights the need for further research in this area.

**Systematic review registration:** [www.crd.york.ac.uk/prospero], identifier [CRD42023409986].

## 1 Introduction

Alzheimer’s disease (AD) is a multifactorial progressive neurodegenerative disorder characterized by memory loss, disorientation, and gradual decline in intellectual ability (Korabecny et al., 2019). It affects approximately 46 million people worldwide and accounts for 60%–80% of all cases of dementia (Ghaffari et al., 2020). The etiology of the disease has not yet been fully elucidated, and only one approved therapeutic approach currently exists for its treatment. The accumulation of beta-amyloid (Aβ) peptides is considered to be one of the fundamental neuropathological pillars of the disease (Dubois et al., 2023), and its dishomeostasis plays a crucial role in its onset (Jeremic et al., 2021; Torres-Mendoza et al., 2022; Tosatti et al., 2022). Researchers are investigating various therapies to combat this disease, including the modulation of targets such as Aβ aggregation, neuroinflammation, and oxidative stress, as well as the use of enhanced multiple biomarkers and risk prediction methods to detect the disease at early stages (Koh et al., 2021; Phadke et al., 2021; Levine et al., 2022). Additionally, research on miRNAs as a possible avenue for AD diagnosis, treatment, and prevention is being conducted (Ghaffari et al., 2020). The efficacy of pharmacological treatment may vary according to the characteristics of different population groups, such as age, disease severity, sex, and presence of other medical conditions (Cardinali et al., 2021).

AD is a progressive neurodegenerative pathology for which there is no definitive cure. However, there are different types of treatments that can help delay its progression and improve the quality of life of affected patients. Treatment options include medication, occupational therapy, cognitive stimulation therapy, physical exercise, massage therapy, music therapy, and nutritional supplements (Pisani et al., 2021). Pharmacological treatments available for AD include donepezil, rivastigmine, galantamine, and memantine (Miculas et al., 2023). These drugs help improve symptoms and delay the progression of the disease in some patients (Rong et al., 2020). Donepezil, rivastigmine, and galantamine are cholinesterase inhibitors used to treat mild to moderate symptoms, whereas memantine is an N-methyl-D-aspartate (NMDA) receptor antagonist used to treat moderate to severe AD symptoms (Birks and Harvey, 2018; Li et al., 2019; Thancharoen et al., 2019). The activity of cholinesterase inhibitors is characterized by the inhibition of the acetylcholinesterase enzyme, responsible mainly for the breakdown of acetylcholine in the nervous system. This allows for the prolonged action of the deficient neurotransmitter in the brain. Rivastigmine has a relatively low protein binding affinity and a more selective action with less possibility of interactions with other drugs. It is important to maintain the balance of different neurotransmitter systems, such as acetylcholine, norepinephrine, dopamine, serotonin, and glutamate, for proper brain function. Although AD is a chronic disease, most research has a limited duration of 6 months, which limits knowledge of the effectiveness of drugs in the long term (Marucci et al., 2021).

Despite advances in available treatments for AD, there is still no definitive cure. Therefore, new treatment approaches are constantly being investigated, such as immunotherapy, gene therapy, light therapy, diet, and physical exercise. Research on new approaches aims to find a more effective and specific therapy than the current treatment options, with the goal of finding a cure for this disease that affects millions of people worldwide (Liang et al., 2018a).

Several psychometric instruments have been used to assess the cognitive and functional performance of patients with AD and other related dementias. The Alzheimer’s Disease Assessment Scale-Cognitive Subscale (ADAS-cog) evaluates memory, attention, reasoning, language, orientation, and praxis (Craft et al., 2020). The Clinical Dementia Rating (CDR) scale measures memory, orientation, judgment and problem-solving, community affairs, home and hobbies, and personal care (Gibson et al., 2020). The Neuropsychiatric Inventory (NPI) assesses a wide range of behaviors in patients with dementia (Gibson et al., 2020). The Alzheimer’s Disease Cooperative Study-Activities of Daily Living (ADCS-ADL) scale is used to evaluate the patients’ performance in basic and instrumental activities of daily living (Gibson et al., 2020). In addition, other global assessment scales, such as the Clinical Dementia Rating-Sum of Boxes (CDR-SB), Clinician’s Interview-Based Impression Plus Caregiver Input (CIBI plus), and Clinical Global Impression (CGI), are used in patients with AD. The Mini-Mental State Examination (MMSE), ADAS-cog, and Severe Impairment Battery (SIB) scales are widely used to evaluate cognition in patients with this disease (Levine et al., 2021).

This study aimed to map out new perspectives on the effect of pharmacological treatment on cognition and overall psychological state in patients with AD.

## 2 Materials and method

### 2.1 Search strategy and data sources

From February 2023 to March 2023, a search was conducted across four databases, (PubMed, Web of Science, Scopus, and the Cochrane Library) to identify documents published within the past 5 years. To achieve the most comprehensive results possible, the search strategy employed was “Alzheimer Disease/drug therapy” [Mesh].

### 2.2 Inclusion criteria

The inclusion criteria for this search were as follows: 1) original articles of randomized clinical trials (RCT); 2) studies conducted in living humans with AD; 3) focusing on cognitive state or psychological aspects, such as memory, mood, etc.; 4) AD must be established at the start of the intervention; 5) adult population over 18 years, both men and women; 6) intervention must be a pharmacological treatment; 7) articles published in English; 8) published within the last 5 years, specifically from 2018 to 2023; 9) with full text available; and 10) methodological quality must score greater than three points on the JADAD scale (Jadad Bechara, 1996).

### 2.3 Exclusion criteria

The exclusion criteria were as follows: 1) studies conducted in animal models, *in vitro*, *in vivo*, and/or post-mortem; 2) studies on biochemical composition or biomarkers; 3) studies on nutritional aspects, or other therapeutic alternatives; and 4) studies on the prevention of AD.

Two researchers conducted the search and screening of the documents, and any discrepancies in the selected documents were resolved through consensus between the researchers. This study was registered in the International Prospective Register of Systematic Reviews (PROSPERO) with the code [CRD42023409986].

## 3 Results

### 3.1 Study characteristics

**FIGURE 1:**
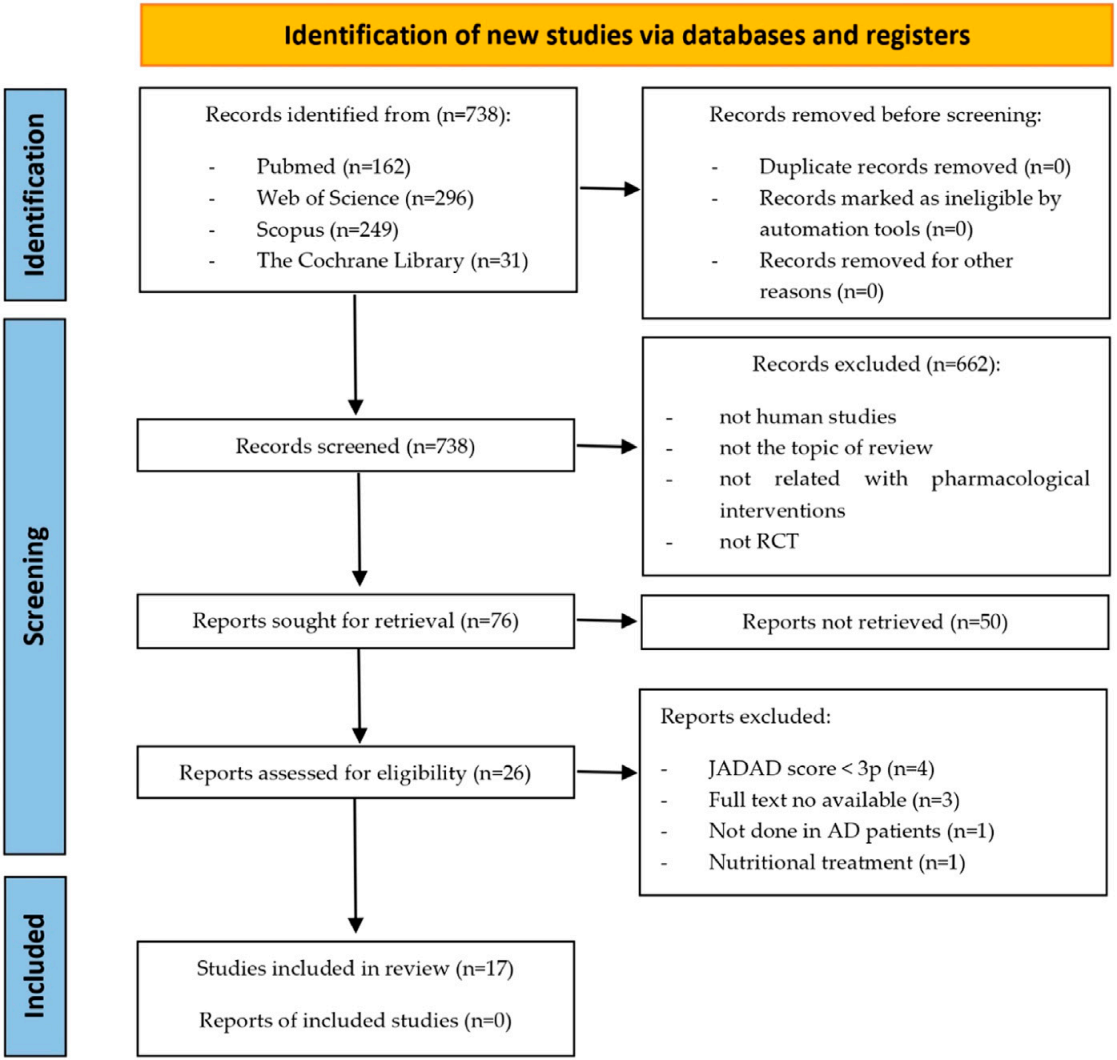
PRISMA flow chart.

A total of 738 documents were initially identified, 17 of which were included in the study. The selection process of the studies included in this review is summarized in Figure 1. The main characteristics of the studies included in this review are presented in Table 1. Additionally, Table 2 displays the JADAD scores of the included articles. Due to the high variability of interventions in the studies included in our systematic review, we have created Table 3 outlining the type of intervention, associated mechanism of action, and effect on cognition. Since the articles included in this systematic review investigate completely different drugs, they have been classified according to their mechanism of action and therapeutic use.

**TABLE 1:** Main characteristics of the studies included in this review.

Reference	Objectives	Sample/Duration	Measuring Instruments	Intervention Design	Results
Craft et al. (2020)	To examine the feasibility, safety, and efficacy of intranasal insulin for the treatment of persons with mild cognitive impairment and AD dementia in a phase 2/3 multisite clinical trial	n = 240 12 months	ADAS-cog-12, ADL-MCI, CDR-SB, blood collection, magnetic resonance imaging	Intranasal insulin 40 UI (n = 121) Placebo (n = 119)	No significant improvement in ADAS-cog-12 nor cerebrospinal fluid outcomes
Dubois et al. (2023)	To evaluate masitinib as an adjunct to cholinesterase inhibitor and/or memantine in patients with mild-to-moderate dementia due to probable AD.	n = 358 24 weeks	ADAS-cog, ADCS-ADL	Masitinib 4.5 mg/kg/day (n = 182) Placebo (n = 176)	*p* < 0.001 ADAS-cog *p* = 0.038 ADCS-ADL
Egan et al. (2018)	To evaluate verubecestat at doses of 12 mg and 40 mg per day, as compared with placebo, in patients who had memory impairment and elevated brain amyloid levels but whose condition did not meet the case definition of dementia	n = 1454 104 weeks	CDR-SB	Verubecestat 12 mg (n = 485) Verubecestat 40 mg (n = 484) Placebo (n = 485)	*p* = 0.67 CDR-SB (Verubecestat 12mg/placebo) *p* = 0.01 CDR-SB (Verubecestat 40mg/placebo). Verubecestat did not improve clinical ratings of dementia. Cognition and daily function were worse in Verubecestat group than in placebo group
Frölich et al. (2019)	To assess the efficacy and safety of BI 409306 at doses of 10–50 mg daily over a 12-week treatment period in two phase II proof-of-concept studies in prodromal and mild AD.	n = 452 12 weeks	NTB, CDR-SB, ADAS-cog11, ADCS-ADL	BI 409306 10 mg QD (n = 77) BI 409306 25 mg QD (n = 74) BI 409306 50 mg QD (n = 76) BI 409306 25 mg BID (n = 76) Placebo (n = 149)	No changes in NTB, CDR-SB, ADAS-cog11, ADCS-ADL
Koch et al. (2020)	To investigate whether therapy with dopaminergic agonists may affect cognitive functions in patients with AD.	n = 94 24 weeks	ADAS-cog, ADCS-ADL, FAB, NPI	Rotigone (n = 47) Placebo (n = 47)	No effect on ADAS-cog. Significant changes in ADCS-ADL and FAB.
Koh et al. (2021)	To assess the effect of GV1001 on the cognition and activities of daily living in patients with moderate-to-severe AD.	n = 96 24 months	SIB, CDR-SB, ADCS-ADL, NPI, MMSE, GDS	GV1001 0.56 mg (n = 33) GV1001 1.12 mg (n = 32) Placebo (n = 31)	GV1001 1.12 mg effectively reduced the change in SIB compared with placebo (*p* < 0.05). Changes in ADCS-ADL and CDR-SB were not significant
Lang et al. (2021)	To determine the effects of 35 mg/day intepirdine *versus* placebo on cognition and activities of daily living in mild-to-moderate AD dementia patients on background donepezil	n = 1315 24 weeks	ADAS-cog, ADCS-ADL	Intepirdine 35 mg/day (n = 661) Placebo (n = 654)	No signficant changes in ADAS-cog (*p* = 0.2249) and ADCS-ADL (*p* = 0.8260)
Lawlor et al. (2018)	To determine if nilvadipine was effective in slowing cognitive decline in subjects with mild to moderate AD.	n = 511 18 months	ADAS-cog12, CDR-SB, DAD	Nilvadipine 8 mg (n = 253) Placebo (n = 258)	No significant changes
Mintzer et al. (2021)	To measure whether methylphenidate compared with placebo decreases the severity of apathy in individuals with AD.	n = 200 6 months	NPI apathy subscale, ADCS-CGIC	Methylphenidate 10 mg (n = 99) Placebo (n = 101)	Significant change in apathy
Padala et al. (2018)	To study the effects of methylphenidate on apathy in AD.	n = 60 12 weeks	Apathy Evaluation Scale-Clinician, MMSE	Methylphenidate (n = 30) Placebo (n = 30)	The methylphenidate group had significantly greater improvement in apathy than the placebo group. There was also greater improvement in cognition, functional status, caregiver burden, CGI scores, and depression in the methylphenidate group compared with the placebo group
Schneider et al. (2019a)	To assess the safety of ladostigil (10 mg/d) and to explore its effect on ameliorating progression from MCI to AD.	n = 210 36 months	NTB, DAD, GDS, CDR, MMSE	Ladostigil 10 mg (n = 103) Placebo (n = 107)	No significant effects on NTB, DAD, or GDS. Ladostigil did not delay progression to dementia
Schneider et al. (2019b)	To assess the efficacy, safety, and tolerability of edonerpic for patients with mild to moderate AD.	n = 484 52 weeks	ADAS-cog, ADCS-CGIC	Edonerpic maleate 224 mg (n = 166) Edonerpic maleate 448 mg (n = 158) Placebo (n = 158)	No clinical effect
Teng et al. (2022)	To evaluate the safety and efficacy of the monoclonal anti-tau antibody semorinemab in prodromal to mild AD.	n = 457 73 weeks	CDR-SB, ADAS-cog13, ADCS-ADL	Semorinemab 1500 mg (n = 94) Semorinemab 4500 mg (n = 136) Semorinemab 8100 mg (n = 92) Placebo (n = 135)	No clinical effect
Vossel et al. (2021)	To determine the ability of the antiseizure drug levetiracetam to improve cognition in people with AD.	n = 34 12 weeks	NIH-EXAMINER, Stroop, ADAS-cog, virtual route learning test, CDR-SB, ADCS-ADL, ADCS-CGIC, NPI	Levetiracetam (n = 17) Placebo (n = 17)	Levetiracetam did not improve cognition function, but improved performance on spatial memory and executive function tasks
Wangchan et al. (2020)	To investigate the long-term therapeutic effects of the Chinese medicine Jiannao Yizhi Formula (JYF) in the treatment of AD.	n = 51 6 months	ADAS-cog, CM-SS, MMSE, MoCA, ADL	JYF (n = 27) Donepezil Control (n = 24)	Compared with baseline, both JYF and donepezil increased the MoCA and MMSE scores and decreased the ADAS-cog and CM-SS scores (*p* < 0.05)
Yang et al. (2019)	To assess the effect and safety of Huannao Yicong Formula (HYF) in the treatment of patients with mild-to-moderate AD.	n = 52 6 months	ADAS-cog, CM-SS, MMSE, MoCA, ADL	HYF (n = 28) Donepezil Control (n = 24)	Compared with the baseline, HYF and donepezil significantly decreased the total scores of ADAS-cog and CM-SS, and significantly increased the scores of MoCA and MMSE (*p* < 0.01)
Yokoyama et al. (2019)	To investigate the therapeutic properties of teprenone in AD.	n = 96 12 months	ADAS-Jcog, MMSE	Donepezil + Teprenone (n = 48) Donepezil + placebo (n = 48)	ADAS-Jcog *p* = 0.861 MMSE *p* = 0.044

AD, Alzheimer’s disease; ADAS-cog, Alzheimer’s disease assessment scale—Cognitive subscale; ADCS-ADL, Alzheimer’s disease cooperative study—Activities of daily living; ADCS-CGIC, Alzheimer’s disease cooperative study—Clinical global impression of change; ADL, Activities of daily living; ADL-MCI, Activities of daily living—Mild cognitive impairment; CDR, Clinical dementia rating; CDR-SB, Clinical dementia rating—Sum of boxes; CM-SS, Chinese medicine symptom scale; DAD, Disability assessment for dementia; FAB, Frontal assessment battery; GDS, Geriatric depression scale; MMSE, Mini-mental state examination; MoCA, Montreal cognitive assessment; NIH-EXAMINER, National institutes of health—Toolbox cognitive battery—Executive function; NPI, Neuropsychiatric inventory; NTB, Neurobehavioral symptom inventory; SIB, Severe impairment battery.

**TABLE 2:** JADAD score of included articles.

	Randomized? yes 1p, not 0p	Method of randomization is adequate? yes 1p, not 0p, not suitable -1p	Double-blind? yes 1p, not 0p	Method of blinding is adequate? yes 1p, not 0p, not suitable -1p	Description of loss of follow-up and abandonments? yes 1p, not 0p	Total score
Craft et al. (2020)	1	1^d^	1	0^b^	1	4
Dubois et al. (2023)	1	1^c^	1	0^b^	1	4
Egan et al. (2018)	1	1^c^	1	1^a^	1	5
Frölich et al. (2019)	1	0^b^	1	1^a^	1	4
Koch et al. (2020)	1	0^b^	1	1^a^	1	4
Koh et al. (2021)	1	1^c^	1	1^a^	1	5
Lang et al. (2021)	1	1^c^	1	1^a^	1	5
Lawlor et al. (2018)	1	1^e^	1	0^b^	1	4
Mintzer et al. (2021)	1	1^c^	0	1^a^	1	4
Padala et al. (2018)	1	1^e^	1	0^b^	1	4
Schneider et al. (2019a)	1	1^e^	1	1^a^	1	5
Schneider et al. (2019b)	1	1^e^	1	1^a^	1	5
Teng et al. (2022)	1	1^e^	1	0^b^	1	4
Vossel et al. (2021)	1	0^b^	1	1^a^	1	4
Wangchan et al. (2020)	1	1^d^	1	1^a^	1	5
Yang et al. (2019)	1	1^d^	1	1^a^	1	5
Yokoyama et al. (2019)	1	1^c^	1	0^b^	1	4

^a^ Identical-appearing tablets

^b^ Not specified

^c^ Computer generated randomization

^d^ Stratified random method

^e^ Block randomization.

**TABLE 3:** Summary of the results obtained in the systematic review.

Type of intervention	Associated mechanism of action	Effect on cognition
Insulin Craft et al. (2020)	Insulin resistance	No effects
Masitinib Dubois et al. (2023)	Inhibitor of tyrosine kinase	Positive effects
GV1001 Koh et al. (2021)	Telomerase	Low effect
Verubecestat Egan et al. (2018)	Inhibitor of BACE-1	No effects
Ladostigil Schneider et al. (2019a)	Inhibitor of MAO	No effects
Semorinemab Teng et al. (2022)	IgG4 antibody	No effects
Rotigotine Koch et al. (2020)	Dopaminergic agonist	Low effect
BI 409306 Frölich et al. (2019)	Inhibitor of PDE9	No effects
Intepirdine Lang et al. (2021)	5-HT6 receptor antagonist	No effects
Nilvadipine Lawlor et al. (2018)	Calcium channel blocker	No effects
Methylphenidate Mintzer et al. (2021)	Increase availability of neurotransmitters	Positive effects
Methylphenidate Padala et al. (2018)	Increase availability of neurotransmitters	Positive effects
Edonerpic maleate Schneider et al. (2019b)	Activation of sigma-1 receptor	No effects
Levetiracetam Vossel et al. (2021)	Positive modulator of SV2A protein	Low effect
Teprenone Yokoyama et al. (2019)	Upregulation of HSP70	Low effect
Jiannao Yizhi Formula Wangchan et al. (2020)	Unknown	Positive effects
Huannao Yicong Formula Yang et al. (2019)	Unknown	Positive effects

### 3.2 Synthesis of clinical trials investigating various treatments for Alzheimer’s disease

#### 3.2.1 Therapeutic potential of anticancer agents in Alzheimer’s disease

Anticancer agents have garnered growing interest in the context of repurposed therapies for AD. To date, results have been controversial. For example, some anticancer drugs such as tyrosine kinase inhibitors and retinoid X receptor agonists can modulate cellular signaling pathways and reduce inflammation, which may be beneficial in the context of AD. Additionally, it has been shown that some anticancer drugs, such as histone deacetylase inhibitors, can improve cognitive function and synaptic plasticity in animal models of AD (Ancidoni et al., 2021). Masitinib is an anti-tumor drug that has been investigated for potential use in the treatment of AD. Its mechanism of action is believed to be related to its ability to inhibit certain enzymes that can contribute to neuronal damage and inflammation in the brain, which in turn may reduce AD symptoms. Masitinib is an inhibitor of tyrosine kinase, an enzyme that plays a role in the activation of inflammatory cells and some brain cells involved in AD progression. By inhibiting this enzyme, masitinib may reduce inflammation and activation of these cells, which could help protect brain cells and reduce AD symptoms (Piscopo et al., 2022; Villain, 2022). Masitinib has demonstrated neuroprotective effects in neurodegenerative diseases by inhibiting mast cell and microglia/macrophage activity and its ability to accumulate in the central nervous system at therapeutically relevant concentrations. Administration of Masitinib at doses of 4.5–6.0 mg/kg/day in patients over the age of 50 with mild to moderate AD-associated dementia has been shown to result in significant improvement in ADAS-cog scores (*p* < 0.001), indicating improved cognition. Masitinib also improved overall function, as assessed by ADCS-ADL (*p* = 0.038). However, this drug has potential safety concerns, including maculopapular rash, neutropenia, and hypoalbuminemia (Dubois et al., 2023). GV1001 is a peptide composed of 16 amino acids corresponding to a fragment of the catalytic site of human telomerase reverse transcriptase. This peptide possesses neuroprotective properties, as it protects neural cells against neurotoxicity, apoptosis, and reactive oxygen species (ROS) induced by Aβ and oxidative stress. These neuroprotective effects are mediated through a variety of mechanisms, such as anti-apoptotic effects, mitochondrial stabilizers, anti-inflammatory, anti-aging, and antioxidant properties. In a clinical study conducted on patients with moderate to severe AD, a dose of 1.12 mg of GV1001 was administered for 24 weeks, resulting in a significant reduction in changes in SIB scores compared to placebo treatment (*p* < 0.05). However, changes in the ADCS-ADL and CDR-SB scores were not significant. Furthermore, GV1001 was well-tolerated without safety issues (Koh et al., 2021).

#### 3.2.2 A novel therapeutic agent for Alzheimer’s disease

The Aβ protein is produced through the sequential action of the β-site amyloid precursor protein (APP) cleaving enzyme (BACE-1) and γ-secretase on the amyloid precursor protein (APP). Inhibition of BACE-1 in preclinical models has been shown to reduce Aβ production and amyloid plaque deposition, which may potentially delay the progression of AD. Verubecestat is a selective inhibitor of BACE-1 that reduces Aβ levels in the cerebrospinal fluid of healthy individuals and AD patients by more than 60% (Egan et al., 2018). However, clinical studies have shown that this inhibitor is ineffective and produces numerous negative side effects, such as rash, dermatitis, sleep disorders, weight loss, and coughing (Miranda et al., 2021). In Egan et al. (2018) RCT, it was found that verubecestat administration for 104 months did not improve clinical dementia symptoms in patients between 50 and 85 years of age. In addition, cognitive and daily function scores were worse in the intervention group than in the placebo group, as measured by the CDR-SB. Adverse effects were also more frequent in the intervention group compared to the placebo group (Egan et al., 2018).

Ladostigil is a novel therapeutic agent that acts as an inhibitor of both monoamine oxidase (MAO) and acetylcholinesterase (AChE) in the brain. It has also been found to possess neuroprotective and antiapoptotic properties by preventing oxidative-nitrate stress and gliosis (Uddin et al., 2020). MAO is an enzyme that degrades important neurotransmitters such as dopamine, noradrenaline, and serotonin. Inhibition of MAO by ladostigil increases the amount of these neurotransmitters in the brain, which could improve cognitive function. Ladostigil also prevents the decline of mitochondrial potential caused by oxidative stress and the release of proinflammatory cytokines from activated microglia. In patients with mild cognitive impairment (MCI), treatment with ladostigil may reduce ROS and proinflammatory changes, suggesting a potential slow action in disease progression. However, in the clinical trial by Schneider et al. (2019b), a dose of 10 mg of ladostigil was found to have no significant effects on cognitive function, daily activity, or depressive symptoms in patients with dementia, as evaluated by NTB, DAD, and GDS, respectively. However, total brain volume and hippocampal volume decreased significantly less in the ladostigil-treated group than in the placebo group, suggesting a potential effect on cerebral atrophy (Schneider et al., 2019a).

Semorinemab is a humanized monoclonal IgG4 antibody that targets the N-terminal domain of tau protein. Its mechanism of action is based on its ability to bind and eliminate Aβ protein fragments, which accumulate as plaques in the brains of patients with AD. Semorinemab was selected for development because of its high affinity and specificity for all known isoforms of full-length tau. Neurofibrillary tangles composed of aggregated tau protein are a hallmark neuropathological feature of AD and are correlated with the clinical severity of the disease. Monoclonal antibodies targeting tau protein may have the potential to slow or stop the spread and accumulation of pathological tau, thereby improving the progression of AD. However, a 73-week treatment with semorinemab in patients with prodromal to mild AD did not result in significant improvements in CDR-SB, ADAS-cog13, and ADCS-ADL. The treatment also did not reduce the rate of cerebral tau accumulation or clinical decline in patients with prodromal-to-mild AD. The safety profile of semorinemab was found to be acceptable and well-tolerated (Teng et al., 2022).

#### 3.2.3 Mitochondrial electron transport inhibitors for Alzheimer’s disease

Dopamine is a crucial neuromodulator that influences several distinct synaptic processes and plays an important role in controlling higher cognitive functions such as memory, learning, and decision-making. Dopaminergic dysfunction may contribute to cognitive impairment in patients with AD. Intervention with a transdermal rotigotine patch, a dopaminergic agonist, did not have an effect on global cognitive dysfunction in patients with mild to moderate AD, as evaluated by the ADAS-cog. However, a significant improvement was observed in the deterioration of activities of daily living, as measured by the ADCS-ADL. This effect appears to be related to minor cognitive dysfunction in the frontal lobe (Koch et al., 2020).

#### 3.2.4 The serotonin 5-HT6 receptor as a therapeutic target for Alzheimer’s disease

One of the key features of AD is an abnormality in glutamatergic neurotransmission related to the function of the N-methyl-D-aspartate (NMDA) receptor in the cortex and hippocampus. Activation of the NMDA receptor signaling pathway produces postsynaptic signaling events through the elevation of second messengers such as cyclic guanosine monophosphate (cGMP). In conditions of NMDA receptor hypofunction, such as in AD, it is hypothesized that inhibition of phosphodiesterase type 9 (PDE9), which hydrolyzes cGMP, may increase cGMP levels and improve NMDA receptor signaling. This could lead to increased plasticity and synaptic stabilization through enhanced long-term potentiation (LTP), thus potentially improving cognitive functions. BI 409306 is a selective and potent inhibitor of PDE9 that has been investigated for the symptomatic treatment of AD. However, administration of this drug at various doses for 12 weeks did not improve NTB, CDR-SB, ADAS-cog11, or ADCS-ADL scores in patients with mild AD. It has not been demonstrated to be effective in improving cognition in patients with prodromal or mild AD (Frölich et al., 2019).

The serotonin 5-HT6 receptor, which is found in critical areas of the brain involved in memory, learning, mood, and behavior, has been studied as a potential therapeutic target for AD. Inhibition of this receptor has been shown to improve the release of important neurotransmitters in AD, which could improve cognition in preclinical models. Specifically, the 5-HT6 receptor antagonist, Intepirdine, has been evaluated in phase 2 clinical trials in AD patients and has been suggested as a possible oral treatment to improve cognition. Since the 5-HT6 receptor is primarily located in the central nervous system, antagonism of this receptor may increase the release of important neurotransmitters and minimize peripheral side effects (Lang et al., 2021; Nirogi et al., 2023). However, Lang et al. (2021) RCT, which evaluated intervention with 35 mg/day of Intepirdine for 24 weeks in patients with mild to moderate AD receiving donepezil as the baseline treatment, did not show significant improvements in ADAS-cog scores (*p* = 0.2249) or ADCS-ADL scores (*p* = 0.8260). Nevertheless, it was observed that Intepirdine demonstrated a favorable safety profile, similar to placebo (Lang et al., 2021).

#### 3.2.5 The neuroprotective mechanisms of calcium channel blockers in Alzheimer’s disease

Nilvadipine is a dihydropyridine calcium channel blocker drug used to treat hypertension (Lawlor et al., 2018; Ling et al., 2021; Dhapola et al., 2022). In addition to its direct blocking action on calcium channels and maintenance of intracellular calcium homeostasis, nilvadipine has been shown to have a number of neuroprotective mechanisms of action. These include reducing the production of amyloid beta 40 and 42 amino acid peptides (Aβ40 and Aβ42) *in vitro* and *in vivo* in transgenic mouse models of AD, and improving Aβ clearance across the blood-brain barrier *in vivo* mouse models (Lawlor et al., 2018). It is believed that these protective effects could have a dual effect on AD pathogenesis, reducing both mitochondrial dysfunction and beta-amyloid accumulation (Ling et al., 2021; Dhapola et al., 2022). However, the results of a RCT conducted by Lawlor et al. (2018) indicate that nilvadipine did not produce significant changes in cognitive decline in patients with mild to moderate AD after receiving 8 mg of nilvadipine for 18 months (Lawlor et al., 2018).

#### 3.2.6 The role of central nervous system stimulants (methylphenidate) treating apathy in AD patients

There are studies suggesting that methylphenidate, a medication used to treat ADHD, may have beneficial effects on patients with AD, such as improving memory and cognition. However, further research is needed to determine its efficacy and safety in individuals with AD. Additionally, it is important to note that methylphenidate is not suitable for all patients and may have side effects. The exact mechanism of action of methylphenidate is not fully known, but it is believed to act by increasing the availability of neurotransmitters such as dopamine and norepinephrine in the brain. These neurotransmitters are involved in the regulation of attention, mood, and cognition. By increasing their availability, methylphenidate may improve attention and memory in patients with ADHD and possibly in those with AD. Furthermore, it is believed that methylphenidate may have neuroprotective effects, protecting nerve cells from oxidative damage and inflammation (Sassi et al., 2020; van Dyck et al., 2021; Andrade, 2022). Regarding apathy in AD, although no treatment has been shown to be effective, catecholaminergic agents such as methylphenidate are promising. It has been proposed that methylphenidate could act as a cognitive enhancer by increasing dopaminergic and noradrenergic neurotransmission, which are diminished in AD (Padala et al., 2018; Mintzer et al., 2021). Although there is still not enough evidence to confirm its efficacy in this regard, studies have shown that intervention with 10 mg methylphenidate for 6 months significantly improved apathy in AD patients, with no significant differences in safety profiles between treatment groups (Mintzer et al., 2021). Additionally, after 12 weeks of treatment with methylphenidate, apathy also significantly improved in patients with mild AD compared to the placebo group, with improvements in cognition, functional status, caregiver burden, CGI scores, and depression (Padala et al., 2018).

#### 3.2.7 The therapeutic effect of neurotransmitter modulators in AD patients

The edonerpic maleate can exert its effects through different mechanisms, including activation of the sigma-1 receptor, modulation of microglial function, and interaction with the collapsin response mediator protein 2 (CRMP2), which facilitates the administration of the AMPA synaptic receptor. According to Schneider et al. (2019a), edonerpic maleate can protect against Aβ-induced neurotoxicity and memory deficits, promote neurite growth, and preserve hippocampal synapses and spatial memory. However, intervention with edonerpic maleate for 52 weeks in patients with mild to moderate AD had no significant effects on the ADAS-cog or ADCS-CGIC scales (Schneider et al., 2019b).

Levetiracetam is an antiepileptic drug that acts as a positive modulator of the SV2A protein, which is a member of the SV2 protein family involved in neurotransmission and is found in most nerve terminals. Levetiracetam binds to the SV2A protein and modulates its function to inhibit neurotransmitter release and reduce neuronal activity (Stout et al., 2019). Decreased levels of SV2A in the brains of patients with AD have been demonstrated, which may contribute to synaptic dysfunction and neuronal loss in AD. SV2 is an important target for new PET tracers that have been developed to visualize synaptic density in the brain (Stout et al., 2019; Carson et al., 2022; Samudra et al., 2023). Although the precise function of SV2 is not fully understood, some possible functions include vesicular transport, stabilization of vesicular neurotransmitter load, anchoring of vesicular proteins, regulation of calcium sensitivity, and interaction with the extracellular matrix. The hypothesis is discussed that SV2 does not directly transport calcium but makes prepared vesicles more sensitive to calcium (Stout et al., 2019). The mechanism of action of Levetiracetam is not fully understood, but it is believed to act by inhibiting the release of glutamate, a neurotransmitter that has been linked to neuronal death in AD. In addition, Levetiracetam has been shown to have neuroprotective effects, helping to prevent brain cell death and neuroinflammation (Musaeus et al., 2021; Vossel et al., 2021). Although Levetiracetam did not improve cognitive function in AD patients in a 12-week intervention study, it improved performance in spatial memory tasks and executive function. It has been studied as a possible treatment to improve cognition in AD patients, but more studies are needed to confirm its effectiveness in this field. Levetiracetam is considered safe and well-tolerated at low doses (Vossel et al., 2021).

#### 3.2.8 Antidiabetics in AD

Currently, research is being conducted to determine the effectiveness of antidiabetic treatments in improving AD due to the possible relationship between type 2 diabetes and AD. Type 2 diabetes is a metabolic disease that affects insulin activity in regulating blood glucose levels. Insulin resistance in the brain, which is an important factor in the development of type 2 diabetes, has also been shown to be related to a higher risk of developing AD (Chen et al., 2022; Malin et al., 2022). Several epidemiological and clinical studies have suggested a possible shared pathophysiology between diabetes and AD, and the administration of certain antidiabetic medications, such as intranasal insulin, metformin, incretins, and thiazolidinediones, has been shown to improve cognition and memory in patients with mild cognitive impairment and AD. As a result, the term “type 3 diabetes” has been proposed for AD, considering it a metabolic disease caused by insulin resistance and insulin-like growth factor in the brain (Čater and Hölter, 2022). Additionally, antidiabetic drugs such as metformin have been observed to improve insulin sensitivity and reduce inflammation in the brain, suggesting that they could slow or reverse the process of cognitive decline in patients with AD. It has been shown that insulin signaling is reduced in the brains of patients with AD, known as cerebral insulin resistance, and is related to the accumulation of beta-amyloid plaques and the formation of neurofibrillary tangles (Michailidis et al., 2022). Although brain glucose metabolism does not depend on insulin, it can alter its use through interactions with the neuronal glucose transporter type 4 (GLUT4) in key cognitive circuits and by promoting glycogen uptake in astrocytes, processes that are considered important during times of high energy demand. Additionally, insulin improves synaptic viability and dendritic spine formation, and modulates levels of key neurotransmitters such as dopamine. Although previous studies have demonstrated that intranasal insulin administration improves performance in the ADAS-cog test and brain glucose metabolism in patients with AD, a randomized clinical trial by Craft et al. (2020) did not find significant effects of intranasal insulin administration on ADAS-cog-12 scores in AD patients, possibly due to the inadequate use of some devices in the study (Craft et al., 2020).

#### 3.2.9 Gastric protectors HSP70 overexpression and teprenone administration as a neuroprotective strategies for AD

It has been reported that elevating HSP70 levels in the brain via genetic modification or teprenone administration in mouse models of AD inhibits the accumulation of Aβ, senile plaque formation, neuronal death, and neurodegeneration, while significantly enhancing memory capacity. Its mechanism of action is believed to involve multiple protective effects, such as reducing Aβ protein production and decreasing cerebral inflammation. HSP70 overexpression leads to positive regulation of Aβ degrading enzyme and TGF-β1 expression, both *in vitro* and *in vivo*. Additionally, teprenone is an antiulcer agent that can inhibit Aβ increase, senile plaque formation, neuronal degeneration, and improve memory. However, a 12-month intervention study in patients with mild to moderate AD who received a combination of donepezil and teprenone did not significantly affect the ADAS-Jcog score (*p* = 0.861), but did impact the MMSE score (*p* = 0.044) (Yokoyama et al., 2019).

#### 3.2.10 Traditional Chinese medicine: a natural alternative for treating Alzheimer’s disease

Traditional Chinese Medicine (TCM) employs a variety of herbal medicines to treat various diseases, including AD, and is considered a natural alternative to synthetic drugs. The mechanisms of action of these herbal medicines have been investigated, and it is believed that they may have beneficial effects in the prevention and treatment of AD. Some herbal medicines may reduce inflammation in the brain, protect nerve cells from damage, and improve cognitive function by modulating different signaling pathways such as NF-κB, Nrf2, JAK/STAT, ubiquitin-proteasome pathway, AMPK/mTOR related to the autophagy-lysosome pathway, GSK-3/mTOR, and PI3K/Akt/mTOR, as well as the SIRT1 and PPARα pathways (Soheili et al., 2021; Ding et al., 2022; Tan et al., 2022). Herbal medicines can also modulate multiple signaling pathways associated with Aβ deposition, protein tau phosphorylation, and chronic inflammation. Some herbal medicines may prevent excessive apoptosis and reduce AChE activity (Fang et al., 2020; Pei et al., 2020; Li et al., 2021). A 6-month study with the Jiannao Yizhi formula increased MoCA and MMSE scores and decreased ADAS-cog and CM-SS scores (*p* < 0.05) in patients with AD. There were no significant differences in the group receiving donepezil, suggesting that the effect of the Jiannao Yizhi formula is not inferior to that of donepezil. The Jiannao Yizhi formula had a favorable safety profile, and no serious adverse effects were found (Wangchan et al., 2020). Similarly, a 6-month study with the Huannao Yicong formula increased MoCA and MMSE scores and decreased ADAS-cog and CM-SS scores (*p* < 0.01) in patients with mild to moderate AD. No serious adverse effects were observed during the study. The Huannao Yicong formula may prevent neuronal apoptosis in the CA1 area of the hippocampus, inhibit secretase activity, and reduce neurotoxicity caused by Aβ peptide in rat models of AD (Yang et al., 2019).

## 4 Discussion

Our systematic review was limited by the scarcity of studies available for discussion of the findings. However, we presented some of the discoveries obtained during the literature search, which could not be included in the systematic review because their failure to meet the established inclusion criteria.

Lithium is considered to be a potential treatment for improving neurotrophic responses and protecting the brain. In patients with amnestic cognitive impairment, treatment with lithium carbonate showed cognitive and functional stability for 2 years, with better performance in memory and attention tests compared to the placebo group (Forlenza et al., 2019). Unlike the expensive aducanumab approved by the FDA in 2021 for patients with mild dementia caused by AD, lithium is more cost-effective and has been shown to be effective for both mild cognitive impairment and AD. In addition, a recent meta-analysis found that lithium is more effective than aducanumab in reducing cognitive decline, as measured by MMSE (Terao et al., 2022). However, some studies indicate that lithium has no significant effect on cognitive performance, and it is important to carefully monitor its administration and follow-up due to its toxicity (Restrepo-Martínez et al., 2022).

Insulin not only regulates glucose homeostasis, but it also has functions in the brain. It has been shown to improve synaptic viability, modulate neurotransmitter levels, such as dopamine, and protect against the toxic effects of Aβ peptide (Craft et al., 2020; Kellar et al., 2022). Unlike the results reported by Craft et al. (2020) (Craft et al., 2020), low insulin levels could be related to AD, and intranasal insulin administration has been shown to improve in cognition in patients with this disease. Intranasal insulin can also reduce the progression of white matter hyperintensity and improve verbal memory (Avgerinos et al., 2018; Zhang et al., 2018; Kellar et al., 2021). Antidiabetic drugs such as intranasal insulin, pioglitazone, rosiglitazone, metformin, sitagliptin, and liraglutide can significantly improve the cognition of patients with AD and mild cognitive impairment; However, metformin does not seem to reduce the risk of AD, and its consumption in the Asian population is associated with a higher risk of this disease, although causality is unknown (Cao et al., 2018; Munõz-Jiménez et al., 2021; Luo et al., 2022).

In the last decade, research has being conducted to evaluate the effect of vaccination and immunotherapy in the treatment of AD (Foroutan et al., 2019; Vaz and Silvestre, 2020). Most of these investigations are in the safety and tolerability phase or in phase I. However, the efficacy of this type of treatment for managing neurodegenerative disease is still unknown (Lacosta et al., 2018). On the other hand, intravenous administration of immunoglobulins has been shown to be ineffective in the treatment of AD, according to reports from previous studies (Okuya et al., 2018; Manolopoulos et al., 2019). Currently, one of the most investigated goals is the treatment of AD with monoclonal antibodies. Despite promising results from clinical trials, the risk-benefit profile of these drugs remains uncertain (Lacorte et al., 2022). Monoclonal antibodies aducanumab and solanezumab have been investigated for their effect on Aβ accumulation and cognitive function. It has been shown that these antibodies can improve cognitive outcomes, evaluated by the ADAS-cog scale (Avgerinos et al., 2021; Budd Haeberlein et al., 2022). Lecanemab (BAN2401) is a humanized monoclonal IgG1 antibody that selectively targets soluble aggregated Aβ species, including oligomers, protofibrils, and insoluble fibrils. Phase II clinical trials have demonstrated that this antibody can reduce brain amyloid burden and clinical decline. However, given that the available evidence is still limited, further studies are needed to fully evaluate its efficacy and safety (Swanson et al., 2021; Dhadda et al., 2022).

Given that pharmacology and nutrition have some points in common, we will also discuss some data we have found regarding the relationship between nutrition and AD. Various nutrients and nutraceuticals have been linked to improvements in cognition and other psychological aspects related to AD (Guzman-Martinez et al., 2021; Abduljawad et al., 2022; Mahnashi et al., 2022; Xu Lou et al., 2023) Some examples include Gingko Biloba (Liao et al., 2020), Melissa Officinalis (Noguchi-Shinohara et al., 2020; Noguchi-Shinohara et al., 2022), Ginseng (Ahmad et al., 2023), anti-inflammatory fatty acids (Albrahim, 2020), medium-chain fatty acids (Juby et al., 2022), ketone bodies (Avgerinos et al., 2020a), saffron (Avgerinos et al., 2020b; Talebi et al., 2021), fenugreek seed (Foroumandi et al., 2023), genistein (Viña et al., 2022), sodium oligomannate (Xiao et al., 2021), anthocyanin (Suresh et al., 2022), microbiota and probiotics (Den et al., 2020; Maitre et al., 2021; Liu et al., 2022; Naomi et al., 2022), benfotiamine (Gibson et al., 2020), omega-3 fatty acids (Canhada et al., 2018; Jernerén et al., 2019), resveratrol (Gu et al., 2021; Buglio et al., 2022; Fang et al., 2022; Tosatti et al., 2022), melatonin (Tseng et al., 2022), citicoline (Bonvicini et al., 2023), folic acid, vitamin B12 (Chen et al., 2021), vitamins and minerals (Mccleery et al., 2018; Karthika et al., 2022), selenium (Pereira et al., 2022), vitamin D (Jia et al., 2019), and mangosteen (Muangpaisan et al., 2022). However, some studies do not support the efficacy of certain nutrients (Zhu et al., 2018; Thancharoen et al., 2019; Araya-Quintanilla et al., 2020; Burckhardt et al., 2020; Du et al., 2020; Prabhakar et al., 2020; Shim et al., 2021; Tofiq et al., 2021; Takada et al., 2022). The effects of these nutrients and micronutrients appear to be related to their anti-apoptotic, antioxidant, and anti-inflammatory properties (de Andrade Teles et al., 2018; Summers et al., 2018; Tamtaji et al., 2019; Zamanian et al., 2021; Rasi Marzabadi et al., 2022). The response to nutritional interventions is greater in the early stages of AD (Moreira et al., 2020), and this response is linked to the APOE genotype (Xu et al., 2020). The maximum benefit of probiotics has been observed in individuals with early cognitive dysfunction and no effect has been found in those with advanced disease or no apparent disease (Akhgarjand et al., 2022; Sánchez-De-Lara-Sánchez and Sánchez-Pérez, 2022). Caprylic acid is a ketone that, when metabolized, produces beta-hydroxybutyrate and acetoacetate ketones, that can cross the blood-brain barrier. Caprylic acid improves cognition in patients with mild-to-moderate AD. This is associated with an increased blood flow in specific brain regions. However, only patients who lack the APOE ε4 allele benefit from this effect (Torosyan et al., 2018).

AD is commonly treated with drugs such as donepezil, rivastigmine, galantamine, and memantine (Li et al., 2019). Although these drugs do not generally cause serious adverse events (Hong et al., 2019), common side effects include headache, diarrhea, nausea, and vomiting (Tricco et al., 2018). Although donepezil is the first-line drug for AD treatment (Birks and Harvey, 2018; Thancharoen et al., 2019), high doses should be administered with caution due to an increased risk of gastrointestinal and cardiac problems (Espiritu and Cenina, 2020; Wang et al., 2022). Both donepezil and memantine are widely used for the treatment of moderate AD (Matsunaga et al., 2018; Rozankovic et al., 2021; Youn et al., 2021). It has been observed that memantine may have a more significant effects on cognition than other commonly used AD medications (Liang et al., 2018b). However, a meta-analysis suggested that the efficacy of memantine is limited in some cases and does not differ significantly compared to placebo (Blanco-Silvente et al., 2018). Although memantine is associated with a lower incidence of AD progression, it also increases the incidence of somnolence (Kishi et al., 2018). Additionally, it is important to note that most available studies have a duration of less than 6 months, and participants usually have mild AD. Optimal pharmacological treatmenst often includes multiple drugs (McShane et al., 2019).

Zolpidem and Zopiclone have been studied for their use in AD patients with insomnia, as insomnia is a frequent problem in these patients (Louzada et al., 2022). Depression is a condition associated with dementia and AD, and sertraline and mirtazapine are antidepressant drugs that have been evaluated in RCTs in patients with AD who also suffer from depression. However, they have not been shown to be effective in these cases (Zuidersma et al., 2019). In contrast, vortioxetine could have beneficial effects on the cognition and mood in elderly patients with AD (Cumbo et al., 2019). While in animal models antidepressants have been shown to delay cognitive decline in animal models, there is still insufficient evidence to support these results in humans (Qin et al., 2022). Agitation and aggression are common symptoms in patients with dementia (Ruthirakuhan et al., 2019; Supasitthumrong et al., 2019), but there are no effective drugs for their treatment. Typical and atypical antipsychotics are commonly used to treat agitation and psychosis in dementia, although their effect on psychosis is insignificant (Mühlbauer et al., 2021). Nabilone may improve agitation; however, more studies are needed to confirm these results (Herrmann et al., 2019). On the other hand, pimavanserin may improve both agitation and aggression in patients with AD (Ballard et al., 2020).

Compared to donepezil, TCM shows no significant difference in effectiveness for treating AD (Hang-kun et al., 2018), although some of its formulas may help improve disease progression (May et al., 2018). For example, Danggui-Shaoyao-San has been found to significantly reduce symptoms in patients with vascular dementia (Kim and Cho, 2020). Furthermore, some studies suggest that a combination of TCM and Western medicine may offer greater benefits than using only one of them (Huang et al., 2021). Traditional Chinese and Japanese medicines have become important sources for drug discovery, and their efficacy in modern drug discovery needs to be investigated (Paudel et al., 2020).

AD is a common type of dementia that has caused a significant global economic and health burden, and there has been a wide debate on the use of statins as a treatment for this disease. Although a systematic review by Mejias-Trueba et al. (2018) did not find statins to improve cognition in AD patients (Mejias-Trueba et al., 2018), a more recent meta-analysis by Xuan et al. (2020) found that statins used in AD patients had beneficial short-term effects on MMSE scores, delaying the deterioration of neuropsychiatric status and significantly improving activities of daily living. However, no benefits were found in the ADAS-cog scores (Xuan et al., 2020).

Animal studies have suggested that TNF-α inhibitors may improve cognition and behavior. However, human studies have been limited (Ekert et al., 2018). Sodium benzoate has been shown to be a cognitive enhancer in patients with AD, schizophrenia, or late-life depression (Lane et al., 2022). On the other hand, estrogen has been shown to delay disease progression and minimize cognitive decline in AD patients, especially in women. However, hormone replacement therapy should be carefully considered due to its potential side effects (Zhou et al., 2020). The new selective glycine transporter-1 inhibitor, BI 425809, has not shown significant clinical improvement in patients with probable AD dementia (Wunderlich et al., 2023). Anti-inflammatory drugs may be beneficial in preventing dementia, although there is no evidence to support the use of aspirin or other NSAIDs (Jordan et al., 2020; Davis et al., 2021). According to epidemiological and laboratory studies, anti-inflammatory drugs may delay or prevent the onset of AD. In observational studies, the use of NSAIDs is significantly associated with a lower risk of AD, especially in long-term users. However, there is no support from RCTs. Neuroinflammation participates in the pathogenic cascades of AD. One possible mode of action for the effectiveness of NSAIDs is through the blocking of COX-2 in the brain. In addition, NSAIDs can also function by activating peroxisome proliferator-activated nuclear receptors, a group of nuclear hormone receptors that act to negatively inhibit the transcription of proinflammatory genes such as IL-6, TNF-α, COX-2, NOS, and cytokines (Wang et al., 2015).

Benzodiazepines and related drugs have been associated with an increased risk of AD in old age and adverse events in patients with mild to moderate AD (Dyer et al., 2020). Eszopiclone may improve sleep quality and cognitive function in elderly patients with AD and sleep disorders (Huo et al., 2022). The combination of various treatments has a better effect than the use of a single treatment or monotherapy in patients with AD, both in moderate and severe stages (Glinz et al., 2019; Guo et al., 2020; Knorz and Quante, 2022). Although our results suggest that methylphenidate has positive effects on apathy associated with AD (Padala et al., 2018; Mintzer et al., 2021), longer follow-up studies are needed to evaluate its efficacy (Lee et al., 2022). Idalopirdine, a selective 5-hydroxytryptamine6 receptor antagonist, has not demonstrated significant efficacy in patients with AD and has been associated with a higher incidence of adverse events (Matsunaga et al., 2019). Current evidence suggests that anti-tau drugs have little impact on slowing cognitive decline (Zheng et al., 2022). The presence of the APOE ε4 genotype, the main genetic risk factor for AD, does not influence the therapeutic effect of acetylcholinesterase inhibitors, but its relationship with other types of drugs is unknown (Cheng et al., 2018). Preclinical studies in transgenic models have suggested that DHP1401 has neuroprotective effects and improves memory. Although studies in humans with this drug are limited (Shim et al., 2022). According to a systematic review by Fink et al. (2018), the use of pharmacological treatments for cognitive protection in individuals with normal cognition or mild cognitive impairment has not been supported. Instead, most studies have been conducted in mild to moderate AD populations, and very few studies have been conducted in more advanced stages (Fink et al., 2018). Verubecestat and lanabecestat have been shown to worsen the cognitive status of patients with AD, although they may improve verbal fluency tasks (Wessels et al., 2020). A relationship has been found between hypertension and an increased risk of AD. Antihypertensive drugs can improve cognition and behavioral symptoms in patients with AD and reduce the incidence of cognitive disorders. Angiotensin receptor blockers are associated with a lower risk of AD, although their potential mechanisms remains unknown (Oscanoa et al., 2021; Rahimi et al., 2021). Epichaperomes play an important role in neuronal pathology, and their inhibition is a promising therapeutic approach for treating neurodegenerative diseases, including AD. However, drugs of this type are still in phase I, and their efficacy is unknown (Silverman et al., 2022). The effectiveness of thiazolidinediones in treating AD is influenced by APOE gene polymorphisms (Iketani et al., 2018). Finally, cannabis-based drugs may inhibit the progression of AD by modulating Aβ modifications. However, more research is needed to determine their efficacy in treating psychiatric manifestations of AD (Farkhondeh et al., 2020; Paunescu et al., 2020).

## 5 Limitations of the systematic review

Although RCTs are methodologically sound according to the JADAD scale, there is still significant variability in the types of interventions used. Only two studies (Padala et al., 2018; Mintzer et al., 2021) have investigated the same active ingredient, highlighting the need for further exploration in this area of research. Additionally, the majority of studies on drugs for AD are conducted in Caucasian populations, despite ethnicity being a factor that can affect treatment efficacy. Ethnic diversity in AD clinical trials remains inadequate (Franzen et al., 2022). Presently, there are no pharmacological therapies that modify the natural progression of AD. Clinical trials in this field typically involve only patients in the early stages of the disease, with those in the advanced stages underrepresented (Ruiz et al., 2022). Symptomatic anti-Alzheimer’s drugs are commonly employed in the treatment of the disease (Watanabe et al., 2019). Overall, our findings suggest that Alzheimer’s pharmacology is a constantly evolving field with significant implications for the development of new pharmacological therapies. We emphasize the critical need for further investigation in this area to advance our understanding of this disease. The findings of our systematic review on the latest advances in Alzheimer’s research indicate that there is still no definitive cure for this disease, at best, its progression can be slowed down. We believe that research into biogenetics and bioengineering could possibly pave the way for new lines of research for the treatment and cure of currently incurable diseases. Many of the drugs included in this systematic review are still in phase III, and therefore, little is currently known about them. Our review opens up new avenues for research into the treatment of AD. While we acknowledge that some of the studies included in our analysis have shown no significant difference between pharmacological intervention and placebo and, in some cases, even worsened the situation, we believe that reporting both positive and negative outcomes is crucial in scientific research to provide a reliable representation of reality.

## 6 Conclusion

In conclusion, although a potential improvement in cognitive function has been observed with some of the evaluated drugs, the limited number of available studies necessitates further research to determine their effectiveness and safety in treating cognitive impairments in Alzheimer’s disease.

## Data Availability

The original contributions presented in the study are included in the article/Supplementary Material, further inquiries can be directed to the corresponding author.
